# ADDRESSING DEMENTIA CLINICAL SYMPTOMS USING NONPHARMACOLOGICAL STRATEGIES: WHAT WORKS AND FOR WHOM?

**DOI:** 10.1093/geroni/igz038.650

**Published:** 2019-11-08

**Authors:** Laura N Gitlin, Laura N Gitlin, Katherine A Marx, Catherine Verrier Piersol, Nancy A Hodgson, Jin Huang, David Roth, Constantine G Lyketsos

**Affiliations:** 1 Drexel University, Philadelphia, Pennsylvania, United States; 2 Johns Hopkins University, Baltimore, Maryland, United States; 3 Thomas Jefferson University, Philadelphia, Pennsylvania, United States; 4 University of Pennsylvania, Philadelphia, Pennsylvania, United States; 5 Johns Hopkins Bayview, Baltimore, Maryland, United States

## Abstract

People living with dementia experience behavioral symptoms and functional decline and their caregivers (CG), reduced wellbeing. In an RCT (N=250 dyads), we tested whether tailoring activities to interests/abilities and providing CGs with instruction in their use (Tailored Activity Program, TAP) reduced clinically significant agitation/aggression (main outcome), functional decline and improved CG wellbeing (secondary outcomes) compared to CG education/support alone; with both groups receiving 8-sessions over 3-months. At 3-months,TAP had no effects on agitation/aggression compared to CG education/support but reduced functional decline (p=0.03), improved CG wellbeing (p=0.01) and confidence using activities (p=0.02). In secondary analyses, black vs. white CGs reported reduced agitation/aggression (p=0.01); female CGs reported reduced burden with TAP whereas male CGs reported reduced burden from education/support (p=0.04); spouses vs. non-spouses reported slower functional decline in participants (p=0.01). This trial suggests outcomes vary by subgroups. Different nonpharmacological approaches are needed for specific clinical characteristics: one size will not fit all.

